# Machine Health Monitoring and Fault Diagnosis Techniques (Volume II)

**DOI:** 10.3390/s24227177

**Published:** 2024-11-08

**Authors:** Shilong Sun, Changqing Shen, Dong Wang

**Affiliations:** 1 School of Mechanical Engineering and Automation, Harbin Institute of Technology, Shenzhen 518055, China; 2 Guangdong Provincial Key Laboratory of Intelligent Morphing Mechanisms and Adaptive Robotics, Shenzhen 518055, China; 3 School of Rail Transportation, Soochow University, Suzhou 215131, China; cqshen@suda.edu.cn; 4 The State Key Laboratory of Mechanical System and Vibration, Shanghai Jiao Tong University, Shanghai 200240, China; dongwang4-c@sjtu.edu.cn

This Special Issue highlights a diverse range of pioneering research dedicated to fault diagnosis, condition monitoring, and defect detection in various engineering systems. Focusing on leveraging state-of-the-art sensor technologies and intelligent diagnostic methodologies, the featured articles collectively aim to enhance the robustness, precision, and efficiency of diagnostic practices.

The collection presents significant strides in the domain, including innovative applications of deep learning algorithms for diagnosing faults in transformers and rolling bearings using vibration signals and time–frequency analyses. For instance, the study titled “*Convolutional Neural Network-Based Transformer Fault Diagnosis Using Vibration Signals*” showcases a convolutional neural network approach that significantly improves fault detection in transformers. Another valuable contribution, “*Bayesian-Optimized Hybrid Kernel SVM for Rolling Bearing Fault Diagnosis*”, presents an enhanced machine learning framework that boosts the accuracy of rolling bearing diagnostics through Bayesian optimization.

Additionally, the research titled “*Research on Diesel Engine Fault Status Identification Method Based on Synchro Squeezing S-Transform and Vision Transformer*” integrates vision transformers with advanced signal transformation techniques, enabling precise fault identification in complex machinery. Another standout paper, “*A New Method for Bearing Fault Diagnosis across Machines Based on Envelope Spectrum and Conditional Metric Learning*”, offers insights into cross-domain diagnostics and demonstrates the adaptability of machine learning models in varied operational environments.

Infrared thermographic (IRT) imaging features prominently within this Special Issue, especially for defect detection in the renewable energy and electronic industries. The review article “*Progress in Active Infrared Imaging for Defect Detection in the Renewable and Electronic Industries*” delves into the use of IRT for high-resolution, non-contact defect detection. This paper examines the integration of IRT with machine learning to identify structural anomalies in photovoltaic panels and electronic components, highlighting practical applications that bolster product quality and reliability.

Furthermore, the paper “*Evaluation of the Diagnostic Sensitivity of Digital Vibration Sensors Based on Capacitive MEMS Accelerometers*” explores digital sensors’ effectiveness in continuous condition monitoring, emphasizing their role in detecting early-stage bearing faults. Complementing this, the study “*Evaluation of Hand-Crafted Feature Extraction for Fault Diagnosis in Rotating Machinery: A Survey*” offers an in-depth analysis of feature extraction methods, balancing computational efficiency and diagnostic accuracy.

In the context of startup conditions, “*Localized Bearing Fault Analysis for Different Induction Machine Start-Up Modes via Vibration Time–Frequency Envelope Spectrum*” investigates fault detection in varying operational states, expanding the understanding of time–frequency signal processing techniques. The article “*A Deep Learning Method for Bearing Cross-Domain Fault Diagnostics Based on the Standard Envelope Spectrum*” highlights the adaptability of machine learning for reliable diagnostics across machine types.

The research “*Prediction of Pre-Loading Relaxation of Bolt Structure of Complex Equipment under Tangential Cyclic Load*” provides a predictive approach to understand and mitigate structural degradation in engineering applications. Additionally, the work “*Preventing Forklift Front-End Failures: Predicting the Weight Centers of Heavy Objects, Remaining Useful Life Prediction under Abnormal Conditions, and Failure Diagnosis Based on Alarm Rules*” offers practical solutions for real-time equipment monitoring and predictive maintenance.

The thorough exploration of these cutting-edge methodologies and applications makes this Special Issue an invaluable resource for professionals, researchers, and engineers dedicated to developing resilient and efficient solutions for equipment reliability and predictive maintenance. The collective expertise of the contributing authors and peer reviewers, alongside the editorial team’s guidance, has enriched this compilation, advancing the pursuit of safer, more reliable, and sustainable industrial practices.

